# Objective Measurement of Physical Activity in Adults With Newly Diagnosed Type 1 Diabetes and Healthy Individuals

**DOI:** 10.3389/fpubh.2018.00360

**Published:** 2018-12-07

**Authors:** Rhys I. B. Matson, Sam D. Leary, Ashley R. Cooper, Catherine Thompson, Parth Narendran, Rob C. Andrews

**Affiliations:** ^1^National Institute for Health Research Bristol Biomedical Research Centre, University Hospitals Bristol NHS Foundation Trust and University of Bristol, Bristol, United Kingdom; ^2^Centre for Exercise, Nutrition and Health Sciences, School for Policy Studies, University of Bristol, Bristol, United Kingdom; ^3^Department of Diabetes, Taunton and Somerset NHS Foundation Trust, Taunton, United Kingdom; ^4^Institute of Biomedical Research, University of Birmingham, Birmingham, United Kingdom; ^5^Department of Diabetes, University Hospitals Birmingham NHS Foundation Trust, Birmingham, United Kingdom; ^6^University of Exeter Medical School, Exeter, United Kingdom

**Keywords:** type 1 diabetes, physical activity, sedentary behaviors, newly diagnosed, moderate-to-vigorous-physical-activity

## Abstract

**Aims:** Physical activity (PA) has many benefits in type 1 diabetes mellitus (type 1 DM). However, PA levels in people with type 1 DM have not previously been measured accurately. We aimed to compare objectively measured PA in adults recently diagnosed with type 1 DM and healthy adults.

**Methods:** Accelerometer data from 65 healthy adults [mean (SD) age 31 (13), 29% men] were compared with data from 50 people with type 1 DM [mean (SD) age 33 (10), 64% men], time since diagnosis <3months, HbA1c 76 ± 25 mmol/mol) in the EXTOD (Exercise for Type 1 Diabetes) pilot study. Briefly, EXTOD investigated the feasibility of recruiting recently diagnosed adults with type 1 DM into a yearlong exercise intervention. Multiple-regression models were used to investigate the association between diabetes status and activity outcomes.

**Results:** Adults recently diagnosed with type 1 DM spent on average a quarter less time in moderate-to-vigorous-physical-activity (MVPA) per day than healthy adults [after adjusting for confounders, predicted values: type 1 DM adults: [mean (SD)] 37.4 mins/day (9.1) Healthy adults: 52.9 mins/day (11.0)]. No difference in MVPA between the groups was seen at the weekend, but adults with type 1 DM spent more time in light physical activity (LPA), and less time in sedentary behavior. Time spent in sedentary or LPA during weekdays did not differ between groups.

**Summary:** Adults recently diagnosed with type 1 DM do less MVPA. Health care workers should encourage these people to engage in more PA. Further studies are needed to assess PA in people with type 1 DM of longer duration.

## Key Messages

- This is the first study to compare objectively measured physical activity in adults newly diagnosed with Type 1 diabetes mellitus (type 1 DM) with people without type 1 DM.
- Adults with newly diagnosed type 1 DM spent over a quarter less time in moderate-to-vigorous-physical-activity (MVPA) than adults without type 1 DM.
- Clinicians should assess activity levels of adults with newly diagnosed type 1 DM and encourage those who are not reaching guideline targets to do more.

## Introduction

Regular physical activity (PA) plays a key role in the management of type 1 diabetes mellitus (type 1 DM) (1). It improves insulin sensitivity and well-being, reduces cardiovascular risk factors such as blood pressure and lipids and may help to preserve beta cell function (2). As a result, guidelines recommend that adults with type 1 DM undertake at least 150 min per week of moderate to vigorous aerobic exercise, spread out over at least 3 days, with no more than two consecutive days between bouts of aerobic activity (3, 4).

Most studies investigating physical activity (PA) levels in adults with type 1 DM have been based on self-reported data rather than objective data. A retrospective analysis of the Diabetes and Complications Trial found 19% of participants (271/1,441) were not achieving recommended PA levels (5). In the EURODIAB prospective cohort study of 2,185 people with type 1 DM from 16 European countries, 786 (36%) people were doing no or only mild PA (6). Similarly, 23% of people with type 1 DM were classed as sedentary and a further 21% were doing less than one session of exercise per week in the Finnish Diabetic Nephropathy Study (7). Only one study has objectively measured PA in adults with type 1 DM. This Canadian study found that only 43% of women and 55% of men with type 1 DM were active (8). No difference was found in activity levels between adults with or without type 1 DM.

In people with established type 1 DM many of the barriers, motivators and facilitators to PA are similar to the general public, such as lack of time, work pressures and bad weather (9–11). They do however require education about the effect of PA on diabetes control to help prevent low and high blood glucose around PA something they struggle to manage and worry about. A qualitative study from our group suggests that newly diagnosed adults with type 1 DM reduce their levels of PA around the time of diagnosis (12). They also face additional barriers to PA such as feeling overwhelmed by their diagnosis and receiving conflicting advice by healthcare professionals to stop exercising.

No studies have objectively measured the PA levels or patterns of people recently diagnosed with type 1 DM, a time when exercise habits may be greatly influenced. This study aimed to compare objectively measured PA levels in recently diagnosed adults with type 1 DM to healthy adults.

## Methods

This is an observational cross-sectional study comparing objectively measured PA data of adults newly diagnosed with type 1 DM with that of healthy adults. The EXTOD study was approved by the Birmingham East, North and Solihull Research Ethics Committee (0/H1206/4), UK, and the study to measure activity in healthy adults was approved by the Centre for Exercise, Nutrition and Health Science Ethics Committee, University of Bristol (EAN 001-15) and was part of RIBM MRes project. All participants provided written informed consent in accordance with the Declaration of Helsinki.

### Recruitment and Procedures

Objectively measured PA in adults with type 1 DM came from participants in the Exercise in type 1 diabetes (EXTOD) study (2). This was a pilot RCT that aimed to assess uptake, intervention adherence, dropout rates and rate of uptake in a usual care group and exercise intervention group over 12-months. This study has been described in detail elsewhere (2, 13). In brief, people aged between 16 and 60 years, clinically diagnosed with type 1 DM in the previous 3-months and self-administering their insulin as part of a multiple dose injection regime, and from 19 UK hospital sites were invited to participate. A member of the clinical team (doctor/diabetes nurse/dietician) at each site approached people newly diagnosed with type 1 DM and obtained written informed consent. Objectively measured PA of participants in this study was measured at baseline, 3, 6, 9 and 12 months. For the present study, the data from the baseline visit were used. Five hundred and eight adults with type 1 DM were invited to take part. Of these 15 took part in the qualitative study and 58 were randomized into the main EXTOD Study (13).

Healthy adults were recruited from the University of Bristol and Taunton and Somerset NHS trust during March-June 2016. A global e-mail was sent out to all members of these two institutions explaining briefly about the study. In addition, flyers were put out in public areas. Participants had to be between 18 and 65 years old and on no medication. Upon request from potential participants, they were sent the participant information sheet, and if interested they were invited to attend an appointment at either the Centre for Exercise, Nutrition and Health Sciences at the University of Bristol or the Diabetes research unit at Taunton and Somerset NHS trust.

### Procedures

Following informed consent, participants completed a questionnaire to confirm their health status, smoking status, and alcohol consumption. Weight and height were measured using standard procedures. BMI was calculated as weight (kg) divided by height (m) squared. Participants were provided with an accelerometer and instructions were given on how to wear the accelerometer for the next 7 days. Participants returned the accelerometer 1 week later either by appointment or by post. Brief feedback was given to the participants on their levels of activity over the 7 days they wore the accelerometer; a graphical interpretation of their physical activity over the 7 days was delivered via email after they had returned the accelerometer and the data had been downloaded.

### Accelerometry

Participants wore the accelerometer (Actigraph Model GT1M or GT3X+; Actigraph LLC, Pensacola, FL, USA) on a belt around the waist during waking hours, apart from swimming or bathing, for 7 days. The accelerometers were set to record data at 30 Hz and data were summarized for every minute. Accelerometer data were downloaded using Actilife software (version 6.11.9, Actigraph LLC) and processed to generate outcome variables using KineSoft (version 3.3.62; KineSoft, Saskatoon, SK, Canada). Non-wear time was defined as a period of 60 min or longer with continuous zero values, with a spike tolerance of 2 min. Thresholds of ≥1,952 counts per minute (cpm) and <100 cpm were used to derive MVPA and sedentary time respectively (14), with light activity defined as 100–1,951 cpm. For a day to be considered valid, a minimum of 480 min (8 h) of wear time was required. For a participant's data to be included in the analysis a minimum of 3 valid days were required.

Outcome measures were time spent in sedentary behavior (minutes per day), time spent in LPA (minutes per day), and time spent in MVPA (minutes per day). Each of these variables were then summarized by weekday, weekend and all week to investigate the pattern of PA throughout the week. The number of bouts of MVPA ≥10 min was also used as a comparison between groups. We used bouts of over 10 min as guidelines suggest that the bouts of exercise that count toward the 150 min a week of moderate or intense activity should be of 10 or more minutes [3.4].

### Analyses

Means/standard deviations and percentages were used to describe the characteristics of the participants. Differences between means of the two groups were tested by two-sample *t*-tests (normal data), Mann-Whitney *U*-tests (non-normal data), and Chi-squared tests (categorical data).

Multiple linear regression was used to assess the association between diabetes status and activity intensity (minutes of sedentary activity, light activity and MVPA). Model A was adjusted for sex, age, wear time, and model B was further adjusted lifestyle factors (BMI, smoking status, and alcohol consumption).

To investigate the difference in the total number of bouts accumulated by both groups, logistic regression was used. Adjustment for confounders (model A and B) was made by creating a binary outcome (equal to or more than the median number of bouts vs. less than the median number of bouts) and fitting multiple logistic regression models. Due to not every participant wearing their accelerometer for 7 days, a sensitivity analysis was performed, which restricted the analysis to only those with 7 valid days of accelerometry.

It had not been appropriate to perform a power calculation for the original study (EXTOD), as it was a pilot. However, a *post-hoc* power calculation showed that with an alpha level of 0.05, a minimum sample size of *n* = 50 in each arm, means of 52 and 37 min/standard deviations of 21 and 28 min of MVPA/day for healthy adults and adults with type 1 DM respectively, this study had 85% power to detect differences between the groups.

All analyses were performed using Stata v15 (*Stata Statistical Software: Release 15*. StataCorp LLC, College Station, TX).

## Results

Fifty-nine participants with type 1 DM had their activity measured at baseline in the EXTOD study and 79 healthy adults were recruited to the study. After excluding participants with invalid accelerometry, there were 50 participants with type 1 DM (85%) and 65 healthy adults (82%) left for analysis. Demographic information for those included in analysis is shown in Table 1. The participants with type 1 DM had a higher percentage of males, higher BMI and a slightly higher amount of accelerometer wear time than the healthy adults but were otherwise similar. Those excluded from analysis were similar to those included in terms of ethnicity, sex, age, smoking status, HbA1c, and duration of diagnosis of type 1 DM, but had lower alcohol consumption and BMI (Supplementary Table 1).

**Table 1:** Characteristics of adults with Type 1 diabetes and healthy adults.

	**Type 1 diabetes (*n* = 50)**	**Healthy adults (*n* = 65)**	***p*-value**
Sex (men) (%)	64%	29%	<0.001
Age (years)	33 (10)	31 (13)	0.6
Ethnicity (% white-british)	85%	85%	0.7
Ex-smoker	20%^a^	20%^b^	0.3
Current smoker	18%^a^	18%^b^	
Never smoked	62%^a^	62%^b^	
Alcohol consumption (units) [Median (IQR)]	3 (10)^a^	6 (7)^b^	0.09
BMI (Kg/m^2^)	24.9 (3.9)	23.4 (3.7)	0.006
HbA1c (mmol/mol) [DCCT (%)]	76.3 (24.6) (9.1%)	–	–
Duration of diagnosis of T1D (days)	62 (23)	–	–
**DAYS OF ACCELEROMETER WEAR (DAYS)**			
Weekday	4.4 (0.8)	4.1 (1.1)	0.07
Weekend	1.6 (0.7)	1.7 (0.5)	0.01
All week	6.0 (1.0)	5.8 (1.5)	0.01

*Mean (SD) unless otherwise stated*.

a *n = 49 for Diabetes*.

b *n = 51 for the healthy adults*.

### Associations Between Diabetes Status and Activity Intensity

Table 2 shows the multiple linear regression models for time spent in sedentary behaviors, LPA, and MVPA in minutes per day.

**Table 2:** Multiple linear regression investigating the associations between diabetes status and activity intensity.

	**Model A**		**Model B**	
**Diabetes vs. no diabetes**	**Regression coefficient (95%CI)**	***p*****-value**	**Regression coefficient (95%CI)**	***p*****-value**
**SEDENTARY**				
Weekday	20.2 (−17.6, 58.0)	0.3	18.0 (−24.6, 60.5)	0.4
Weekend	−19.3 (−56.7, 18.1)	0.3	−40.6 (−82.1, 0.9)	0.06
All week	9.9 (−23.4, 43.2)	0.6	3.5 (−34.3, 41.4)	0.9
**LIGHT**				
Weekday	−3.8 (−39.1, 31.5)	0.8	−3.7 (−44.7, 37.3)	0.9
Weekend	35.2 (3.3, 67.0)	0.03	48.3 (12.5, 84.0)	0.009
All week	5.1 (−25.4, 35.6)	0.7	7.2 (−27.8, 42.2)	0.7
**MVPA**				
Weekday	−16.7 (−27.5, −6.0)	0.003	−13.5 (−25.5, −1.5)	0.03
Weekend	−15.8 (−31.6, 0.07)	0.05	−7.5 (−25.4, 10.4)	0.4
All week	−15.2 (−25.2, −5.1)	0.003	−10.9 (−22.2, 0.4)	0.06

*All week and weekday sedentary time, light physical activity (LPA) and moderate-to-vigorous-physical-activity (MVPA): Model A n = 115 Model B n = 99. Weekend sedentary, light and MVPA: Model A n = 107 and Model B n = 91. Model A adjusts for sex, age, and wear time. Model B adjusts for sex, age, wear time, BMI, smoking status, alcohol consumption*.

For time spent in sedentary behaviors and LPA, there was no evidence of a difference on weekdays and over the whole week, but there was evidence for the adults with type 1 DM spending more time in LPA and less time in sedentary behaviors at the weekend.

For MVPA, statistical evidence was found for a difference on weekdays and across the whole week, but not on weekends. On average the type 1 DM group spent 10.9 min less in MVPA per day than the healthy adults after adjustment for confounders [(95% CI-22.2, 0.4) *p* = 0.06], over a quarter less time in MVPA [Predicted values [Mean (SD)] type 1 DM adults: 37.4 mins/day (9.1) Healthy adults: 52.9 mins/day (11.0)].

### Associations Between Diabetes Status and Bouts of MVPA

We investigated the difference in number of bouts of MVPA ≥10 min further by logistic regression, using the binary variable based on the median number of bouts as the outcome. No statistical evidence was found for a difference in numbers of bouts between the groups for either models (Supplementary Table 2).

## Discussion

We have shown for the first time that adults recently diagnosed with type 1 DM spent over a quarter less time doing MVPA over the week than healthy adults. This difference seemed to be driven by the weekdays rather than weekend. There was also evidence that people with type 1 DM spent more time in LPA and less time being sedentary than healthy adults at the weekend, but no differences in these behaviors was seen on weekdays.

We could only find two studies that have compared objectivity measured physical activity of people with type 1 DM with healthy adults. In the first study Brazeau and colleagues using a motion sensor (SenseWear Pro 3 Armband) measured the activity of 75 Canadian adults with established type 1 DM and 75 Canadian adults without type 1 DM (8). They found that although the PA levels of the adults with type 1 DM were low, only 43% of women and 55% of men being classed as being active, they were not different from those seen in adults without type 1 DM. The difference between our finding and the findings of this study could be due to the fact that our participants were adults with newly diagnosed diabetes whereas in the Brazeau et al. study the participants had had type 1 DM on average for 23 years. In a qualitative study that our group conducted of newly diagnosed people with type 1 DM around half reported a reduction in activity levels around diagnosis (12). In addition, some participants reported being advised by healthcare practitioners not to exercise. Finally, adults newly diagnosed with type 1 DM placed greater emphasis on fear of hypoglycaemia than previous studies of people with longstanding type 1 DM (11).

In the second study, by Finn and colleagues, PA was measured objectivity using an accelerometer (Actigraph) in 72 (34 males) Irish adults with type 1 DM (15). Subjectively reported PA levels were also captured using the International Physical Activity Questionnaire (IPAQ). The mean age (± SD) was 41 (± 13) years and mean diabetes duration of 18 (± 12) years. This study found 23 (32%) participants exercised to PA recommendations as measured by accelerometry, compared with 69 (97%) participants reporting meeting the recommendations as per the IPAQ. This study as well as confirming that people with type 1 DM are not very active also underlined the inaccuracy of physical activity questionnaires and the need to use objective measures of activity.

It is interesting that the lower MVPA in adults with type 1 DM appears to be driven by changes in weekday activity rather than weekend activity. During the week activity is influenced by work patterns and the type of job a person does whereas at the weekend people tend to have more control over their activity patterns. It might well be that the difference we are seeing in this study are due to a difference in the way that people travel to work (walking vs. driving car) (16). We were unable to look at this, as occupation was not recorded. Alternatively, people newly diagnosed with type 1 DM may have had restrictions placed on them by their work due to their diagnosis that may have reduced their levels of activity. This is not something that came up in our interviews with newly diagnosed people with type 1 DM (12) but is something that people on occasions have mentioned to us in clinic. Studies are needed to confirm whether this does happen and if so how often. People with type 1 DM often have to plan their exercise as they may have to make a change to their insulin dose at the meal prior to exercise or eat before exercise. This may mean that they tend to focus on exercise at the weekend rather than during the week when they have more time to plan for exercise. People with type 1 DM can have problems with low or high blood glucose for 24 h after exercise. Because they do not want to have these problems on a working day, they might decide to do their exercise at the end of the week and at the weekend.

More time spent being sedentary is associated with poorer metabolic health. In a systematic review of 29 observational studies, accelerometer-measured total sedentary time was detrimentally associated with both glycaemic control and lipid profile (17). People with newly diagnosed type 2 diabetes mellitus as well as doing less MVPA than healthy controls spend more time being sedentary (18). Conversely, in the present study adults newly diagnosed with type 1 DM were not found to be more sedentary than healthy controls. If this finding is replicated in other studies, then exploring why this is the case might give us better insight in how to promote higher levels of MVPA and reduce sedentary time.

The lack of difference in sedentary time could be due to the fact that although their worries about hypoglycaemia, and lack of knowledge and/or confidence in managing their glucose around exercise prevents them from doing moderate and high intensity activities it does not stop them doing low intensity activities. Alternatively, it could be that a reduction in sedentary time is only seen after people have had type 1 DM for a number of years. Brazeau and colleagues did not report sedentary time in their study (8). In the study by Finn and colleagues time spent in sedentary time was high, with the mean time spent being sedentary 8.4 ± 1.6 h per day (15), but as they did not have healthy controls it is difficult to know if this is more than that seen in people without type 1 DM of similar age and weight. Further studies will be needed to clarify how sedentary people with type 1 DM are and whether this changes with time.

In our data set, women were less active than men (data not shown). This is in keeping with an American study that objectively measured PA in 6,329 participants and found men to be more active than women (19) but at odds with objectively measured activity data from 93,015 participants in the UK Biobank study where no difference in activity was seen between men and women (20). In our analysis we have tried to adjust for the fact that there were more men in the Type 1 group but we might have found a greater difference in MVPA if our two groups had been equally matched for men and women.

### Strengths and Limitations

A major strength of this study is the use of an objective and reliable method to assesses the physical activity levels (21). In addition, people with type 1 DM came from the EXTOD study (2) which recruited from multiple UK sites covering both large teaching and district general hospitals, and participants spanned a wide age range. However, there are limitations to this study. Due to the fact that we only measured activity at one time point, we are unable to comment on any causal associations between recent diabetes diagnosis and changes in PA. Healthy controls were recruited from only two sites both in the South West of the UK, which may limit generalizability. It is also likely that study participants were more interested in exercise than those who declined, and PA may be lower in the general population of both type 1 DM and healthy people. The small sample size of the two groups in this study is a further limitation of this study. Nonetheless, this study adds to the literature suggesting that longitudinal observational studies of PA of people with newly diagnosed type 1 DM are warranted.

## Conclusion

In conclusion, our results indicate that adults newly diagnosed with type 1 DM spend more than a quarter less time doing MVPA over the week compared to healthy adults. This suggests that steps need to be taken to try and improve the activity levels of adults newly diagnosed with type 1 DM. To do this clinicians will need to be trained in how to assess and encourage activity and also be furnished with knowledge about how to advise patients to manage their glucose around exercise. In addition, people with type 1 DM will need to be provided with knowledge and skills to safely manage their glucose around exercise. We and others are working on programmes to help support these changes.

## Author Contributions

RM, SL, AC, and RA had full access to the primary data. All authors contributed to data analysis and interpretation, and the writing and editing of the report. All authors approved the final version of the report. The corresponding author had full access to all the data in the study and had final responsibility for the decision to submit for publication.

### Conflict of Interest Statement

RA has received honoraria from Novo Nordisk, Sanofi-Aventis, and Merck Sharp &amp; Dohme, and travel expenses from Sanofi-Aventi. PN has received honoraria and travel expenses from Novo Nordisk, Sanofi-Aventis and Lilly. The remaining authors declare that the research was conducted in the absence of any commercial or financial relationships that could be construed as a potential conflict of interest.

## Abbreviations

Type 1 DM: type 1 diabetes mellitus
PA: Physical activity
LPA: Light physical activity
MVPA: moderate-to-vigorous-physical-activity.
