# Supplementary material for: Objective Measurement of Physical Activity in Adults With Newly Diagnosed Type 1 Diabetes and Healthy Individuals
Source: Front Public Health. 2018 Dec 7;6:360. doi: 10.3389/fpubh.2018.00360 (PMC6293090; doi:10.3389/fpubh.2018.00360)
Supplement: Supplementary file 1 [file Table_1.DOCX]

**Appendices**

| **Supplementary Table 1-** Characteristics of adults with Type 1 diabetes and healthy adults excluded vs included | | | | |
| --- | --- | --- | --- | --- |
|  | Excluded (n=23) | Included (n=115) | p-value |  |
| Gender (male) (%) | 35% | 44% | 0.5 |  |
| Age (Years) | 32.7 (12.0) | 31.8 (12.0) | 0.4 |  |
| Ethnicity (% White-British) | 87% | 86% | 0.4 |  |
| Ex-smoker  Current smoker  Never smoked | 6.5%^a^  6.5%^a^  87%^a^ | 14%^c^  17%^c^  69%^c^ | 0.4 |  |
| Alcohol consumption (units) (Median (IQR)) | 1 (5)^a^ | 5 (9)^c^ | 0.02 |  |
| BMI (Kg/m^2^) | 22.4 (2.6) | 24 (3.9) | 0.05 |  |
| HbA1c (mmol/mol) (DCCT (%)) | 66.1 (23.8)^b^ (8.2%) | 76.3 (24.6)^d^ (9.1%) | 0.3 |  |
| Duration of diagnosis of T1D (days) | 68.1 (22.8)^b^ | 62.2 (23.0)^e^ | 0.5 |  |

Mean (SD) unless otherwise stated.  ^a^n=15 ^b^n=8 ^c^n=99 ^d^n=49 ^e^50.

| **Supplementary Table 2 -** Association between diabetes status and number of bouts of MVPA ≥10 minutes | | | | |
| --- | --- | --- | --- | --- |
|  | Model A |  | Model B |  |
| Diabetes vs no diabetes | Regression coefficient (95% CI) | p-value | Regression coefficient (95% CI) | p-value |
| Weekday | 0.7 (0.3, 1.6) | 0.4 | 0.9 (0.3, 3.1) | 0.9 |
| Weekend | 1.0 (0.4, 2.5) | 0.96 | 1.3 (0.3, 3.9) | 0.8 |
| All week | 0.6 (0.3,1.5) | 0.3 | 1.0 (0.3, 3.5) | 0.996 |

Logistic regression using median number of bouts as the cutoff. All week and weekday n: model A= 109 model B= 93. Weekend n: model A=104. Model B=88. Model A adjusts for age, gender and wear time. Model B adjusts for age, gender, wear time, smoking status, alcohol consumption and BMI.
